# Functional characterization and anti-cancer action of the clinical phase II cardiac Na^+^/K^+^ ATPase inhibitor istaroxime: *in vitro* and *in vivo* properties and cross talk with the membrane androgen receptor

**DOI:** 10.18632/oncotarget.8329

**Published:** 2016-03-24

**Authors:** Konstantinos Alevizopoulos, Konstantinos Dimas, Natalia Papadopoulou, Eva-Maria Schmidt, Anna Tsapara, Saad Alkahtani, Sabina Honisch, Kyriakos C. Prousis, Saud Alarifi, Theodora Calogeropoulou, Florian Lang, Christos Stournaras

**Affiliations:** ^1^ Pharmacellion Ltd, CH61 9PN, Wirral, United Kingdom; ^2^ Laboratory of Pharmacology, Faculty of Medicine, University of Thessaly, Larissa, Greece; ^3^ Department of Biochemistry, University of Crete Medical School, Heraklion, Greece; ^4^ Department of Physiology, University of Tübingen, Tübingen, Germany; ^5^ Department of Zoology, Science College, King Saud University, Riyadh, Saudi Arabia; ^6^ Institute of Biology, Medicinal Chemistry and Biotechnology, National Hellenic Research Foundation, Athens, Greece; ^7^ Department of Medical Psychology and Behavioral Neurobiology, University of Tübingen, Tübingen, Germany

**Keywords:** Na^+^/K^+^ ATPase, istaroxime, prostate cancer, actin cytoskeleton, membrane androgen receptor

## Abstract

Sodium potassium pump (Na^+^/K^+^ ATPase) is a validated pharmacological target for the treatment of various cardiac conditions. Recent published data with Na^+^/K^+^ ATPase inhibitors suggest a potent anti-cancer action of these agents in multiple indications. In the present study, we focus on istaroxime, a Na^+^/K^+^ ATPase inhibitor that has shown favorable safety and efficacy properties in cardiac phase II clinical trials. Our experiments in 22 cancer cell lines and in prostate tumors *in vivo* proved the strong anti-cancer action of this compound. Istaroxime induced apoptosis, affected the key proliferative and apoptotic mediators c-Myc and caspase-3 and modified actin cystoskeleton dynamics and RhoA activity in prostate cancer cells. Interestingly, istaroxime was capable of binding to mAR, a membrane receptor mediating rapid, non-genomic actions of steroids in prostate and other cells. These results support a multi-level action of Na^+^/K^+^ ATPase inhibitors in cancer cells and collectively validate istaroxime as a strong re-purposing candidate for further cancer drug development.

## INTRODUCTION

Na^+^/K^+^ ATPase is a well-established pharmacological target acting as a receptor for cardiotonic steroids (CTS) such as digoxin and digitoxin [1, 2]. Most recently, a new generation of non-sugar containing steroidal enzyme inhibitors with improved therapeutic indices (i.e. better ratio of therapeutic activity versus toxicity) has been reported [3–7]. Istaroxime represents the lead candidate from this class and has successfully concluded phase II clinical trials in cardiac failure patients [8–10]. Na^+^/K^+^ ATPase is also emerging as a novel anti-cancer target. Overall, four layers of evidence support this role (reviewed in [11]). First, aberrant expression of enzyme subunits has been observed in a growing number of cancers. Second, several different CTS have shown outstanding activities in chemical and functional screens. Third, data from multiple epidemiological studies involving CTS-treated cardiac patients have shown reduced cancer development risk, incidence or mortality in certain cancer indications. Finally, clinical trials with various CTS have proven the promising anti-cancer potential of these inhibitors.

Based on the promising anti-cancer properties described above, our group previously assessed the anti-cancer potential of novel Na^+^/K^+^ ATPase inhibitors [3–7], showing strong activity of these compounds in multiple cancer cell lines [12, 13]. Moreover, 3-R-POD, the most active derivative of this series, exhibited dose dependent tumor inhibition in prostate and lung cancer xenografts *in vivo* [12, 13]. In the current study, we focused on istaroxime, the lead inhibitor of this class. Specifically, we show that istaroxime is active in 22 different cancer cell lines derived from 9 tumor panels *in vitro* as well as in prostate cancer xenografts *in vivo*. We demonstrate novel mechanistic insights on the signaling of this compound in prostate cancer cells. Moreover, we link for the first time Na^+^/K^+^ ATPase and mAR, a membrane androgen receptor mediating rapid, non-genomic anti-cancer effects of androgens in multiple cancer cells [14, 15]. Our results provide novel insights into the anti-cancer properties of istaroxime further supporting development of this agent as a novel anti-cancer drug candidate.

## RESULTS

### *In vitro* anti-cancer activity of istaroxime in multiple cell lines

**Figure 1:**
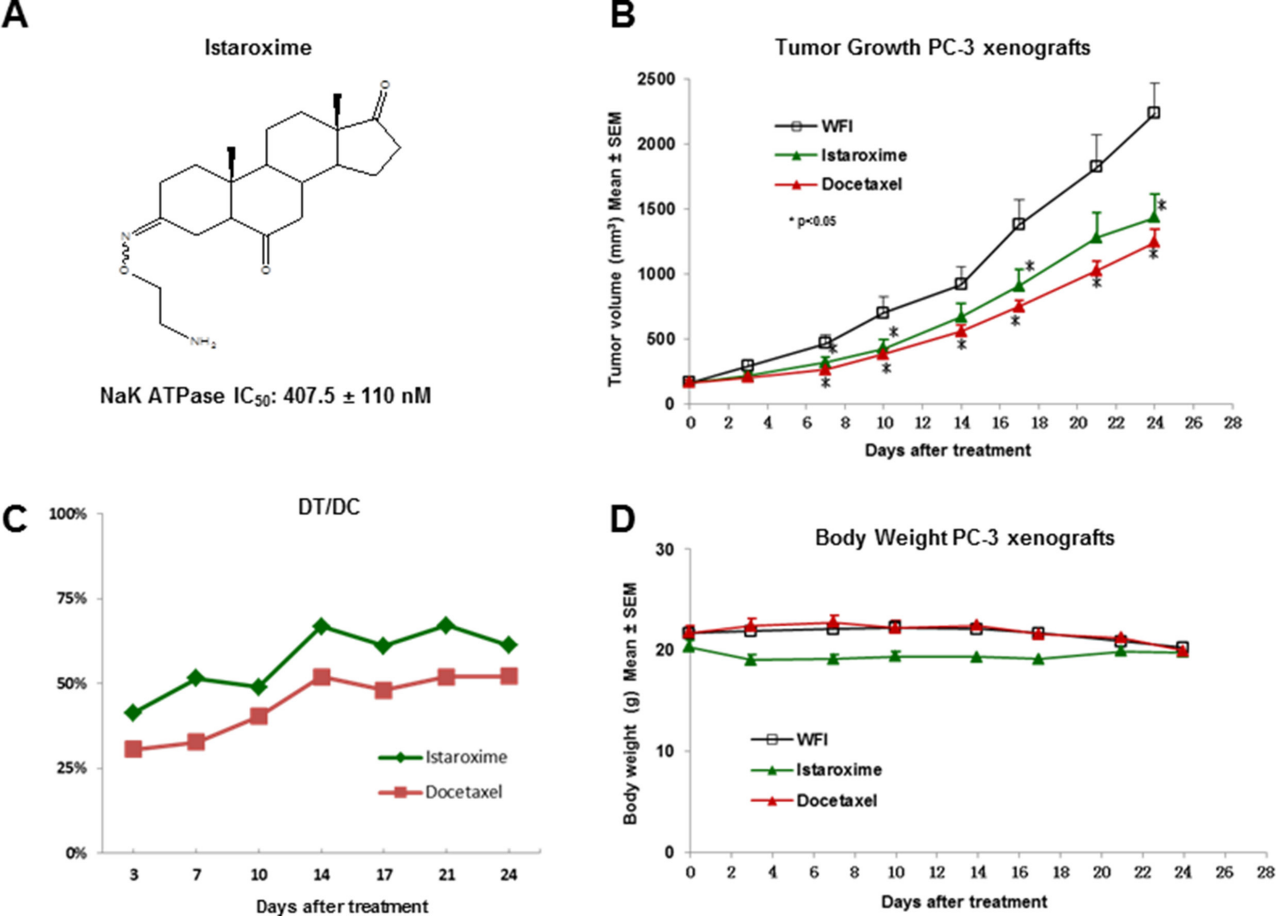
Anti-cancer activity of istaroxime in PC-3 prostate cancer xenografts (**A**) Chemical structure of istaroxime ((E, Z) 3-(2-aminoethoxyimino)-5α-androstane-6, 17-trione). The IC_50_ inhibitory activity of the compound was determined at 407.5 ± 110 nM (*n* = 4); this value is comparable to the published IC_50_ value (430 ± 115 nM) of the compound [6]. (**B**) Tumor size of animals treated with istaroxime (22.5 mg/kg) administered IP, twice daily versus docetaxel (12 mg/kg) dosed intravenously, once weekly, and vehicle control (WFI: water for injection). *indicates dose dependent statistically significant anti-cancer activity versus control (ANOVA, *p* < 0.05, days 7–10, 17–24). (**C**) DT/DC measurements for istaroxime and docetaxel calculated as described in Methods. (**D**) Body weight of mice treated by different compounds as described in panel B.

Having recently characterized 17 cardiac enzyme inhibitors in anti-cancer assays [12, 13], we tested the anti-cancer activity of istaroxime, the prototype cardiac inhibitor of this class (Figure 1A, Na^+^/K^+^ ATPase IC_50_: 407.5 nM). Specifically, we determined GI_50_, TGI and LC_50_ values (see SRB assays, Methods) of the compound in 22 different cancer cell lines from 9 tumor panels (lung, melanoma, ovarian, renal, CNS, breast, pancreas, colon and prostate). Istaroxime exhibited GI_50_ and LC_50_ values in the low micromolar range in all cell lines; PC-3 and DU145 prostate cancer cells were among the most sensitive cells to the action of the compound (Table 1). Although some anti-proliferative activity was observed in normal fibroblasts, TGI and LC_50_ values of istaroxime were significantly higher in comparison to values in cancer cells (Table 1). Interestingly, and as shown previously for other inhibitors of the same class [12], istaroxime exhibited comparable anti-cancer activity in multi-drug resistant NCI/ADRRES cells [16, 17]. Similar results were observed with MTT assays in DU145 and CAKI-1 cells (Table 1) further confirming istaroxime's anti-cancer action.

**Table 1:** SRB and MTT assays performed with istaroxime

SRB	A549	EKVX	NCI-H460	SKMEL28	LOXIMVIb	MDA-MB-435	CCSWD1	MCF7
(μΜ)	Lung	Lung	Lung	Melanoma	Melanoma	Melanoma	Melanoma	Breast
**GI_50_**	3.59	3.43	3.21	8.15	6.72	6.43	3.43	2.45
**TGI**	6.56	6.70	6.10	53.91	38.37	38.66	6.15	5.56
**LC_50_**	9.53	9.96	8.99	> 100	> 100	> 100	8.87	8.67

SRB	T47D	OVCAR-5	OVCAR-3	IGROV1	NCI-ADRRES	DU145	PC3	SU8686
(μΜ)	Breast	Ovarian	Ovarian	Ovarian	Ovarian	Prostate	Prostate	Pancreas
**GI_50_**	4.91	5.59	5.20	4.45	3.45	3.33	1.72	3.30
**TGI**	8.61	12.27	40.28	8.92	6.01	6.01	5.01	6.02
**LC_50_**	> 100	80.54	> 100	> 100	8.57	8.69	8.30	8.74

SRB	CAKI-1	SF-295	U251	SF-268	HCT-116	HCT15	Fibro	
(μΜ)	Renal	CNS	CNS	CNS	Colon	Colon	Dermal	
**GI_50_**	3.28	3.01	3.34	2.30	3.19	3.20	7.1	
**TGI**	6.50	5.71	6.16	5.60	7.45	6.37	85.1	
**LC_50_**	9.72	8.41	8.97	8.89	60.77	9.53	> 100	

MTT	DU145	CAKI-1						
(μΜ)	Prostate	Renal						
**IC_50_**	2.28	3.21						

SRB columns: calculated GI_50_, TGI and LC_50_ values of istaroxime in SRB assays performed in 22 cancer cell lines from 9 tumor panels. Fibro: human dermal fibroblasts. MTT columns: calculated IC_50_ values in MTT assays performed in DU145 and CAKI-1 cells. Values with “>” symbols: calculated values superior to the depicted value. Results are average values from 2–3 independent experiments. All values are in μM.

### Istaroxime shows anti-cancer activity in PC-3 prostate cancer xenografts

To further characterize the anti-cancer properties of istaroxime, we have performed experiments in PC-3 prostate xenografts *in vivo*. Initially, we determined the compound's maximum tolerated dose (MTD) by injecting increasing doses in NOD/SCID mice via intraperitoneal (IP) injection. These studies defined the acute MTD (single dose) at 200 mg/kg. Daily administration of 40–50 mg/kg over a period of several days (chronic administration) was also well tolerated (data not shown). Taken together this dosing information and the short half-life of the compound (< 1 hour; [18]), we have selected a dose of 22.5 mg/kg administered IP twice daily (at 12 hours intervals) for prostate xenograft experiments. Docetaxel, an anti-cancer drug approved for metastatic prostate cancer was also included as a positive control in our assays (dosed intravenously at 12 mg/kg, once weekly). As shown in Figure 1B, istaroxime showed statistically significant tumor growth inhibition against PC-3 xenografts (*p* < 0.05, days 7, 10, 17 and 24). DT/DC values ranged between 41–67% throughout the experiment (Figure 1; Panel C), whereas Tumor Growth Inhibition (TGI) at day 24 was 43.1%. Neither istaroxime nor docetaxel significantly modified body weight as indicator of toxicity (Figure 1; Panel D). Similar results were obtained in a separate xenograft experiment employing a dose of 40 mg/kg injected IP once daily (four daily treatments followed by three days of rest for three weeks; data not shown). Altogether, these results confirm the anti-cancer activity of istaroxime in prostate cancer xenografts *in vivo*.

### Istaroxime induces apoptosis and caspase-3 activation in prostate cancer cells

**Figure 2:**
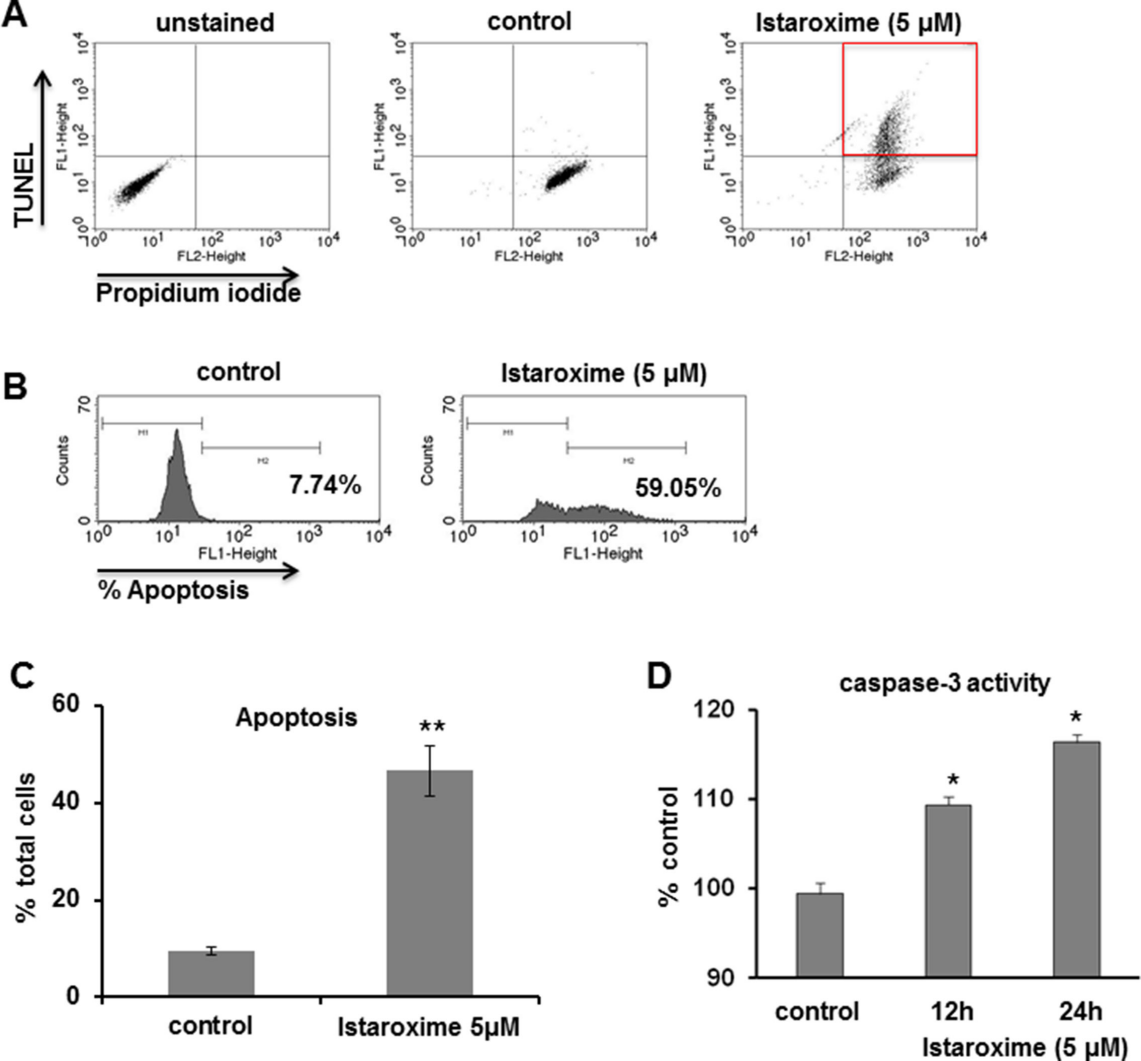
Istaroxime induces apoptosis and caspase-3 activation in prostate cancer cells (**A–C**) DU145 prostate cancer cells were treated for 24 hours in serum-supplemented conditions in the absence (control) or presence of 5 μΜ istaroxime. Apoptosis was measured using the APO-BrdU^™^ TUNEL Assay kit as described in Methods. Final detection of BrdU incorporation at DNA break sites is achieved using an Alexa Fluor488–labeled anti-BrdU antibody thus identifying TUNEL positive cells, while Propidium Iodide is used to stain the total cell population. Apoptotic cell population was determined by FACS analysis. (A and B) FACS analysis of a representative experiment shows the percentage of TUNEL positive cells in control and istaroxime treated cells after measuring 10.000 cells. (C) Quantification of apoptotic response in control and istaroxime-treated cells. Data presented in bars are mean values ± SE of *n* = 3 independent experiments (**P < 0.01). (**D**) Caspase-3 activity was measured at 405 nm in lysates derived from cells exposed to 5 μΜ istaroxime for the indicated time periods and then incubated with the caspase-3 substrate DEVD conjugated to the chromophore pNA as described in Methods. The relative caspase-3 activity is expressed as percentage with that of serum cultured cells taken as 100%. Data presented in bars are mean values ± SE of *n* = 6 independent experiments (*P < 0.05).

We further analyzed the apoptosis of DU145 prostate cancer cells treated with 5 μM of the compound over a period of 24 hours. As shown by FACS analysis (Figure 2, Panels A, B), istaroxime increased the number of apoptotic cells from 9.48% in control samples to 46.54% following treatment (Figure 2, Panel C). In agreement with the FACS data, istaroxime induced a modest, yet reproducible increase in caspase-3 activity peaking at 24 h post treatment initiation (Figure 2; Panel D).

### Istaroxime reduces c-Myc expression and induces actin cytoskeleton re-organization and RhoA activation in prostate cancer cells

**Figure 3:**
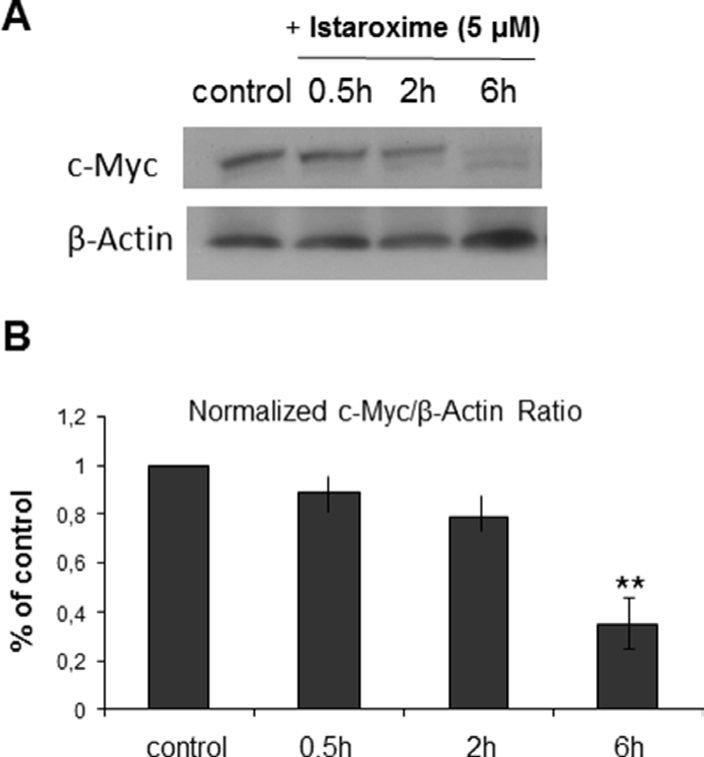
Istaroxime reduces c-Myc expression in prostate cancer cells (**A**) Representative blot from *n* = 3 experiments showing c-Myc protein expression in DU145 prostate cells untreated (control) or treated with 5 μΜ istaroxime for 0.5, 2 and 6 hours. (**B**) Bars represent normalized expression of c-Myc / ß-Actin ratio as percentage of control (*n* = 3, **P < 0.01).

**Figure 4:**
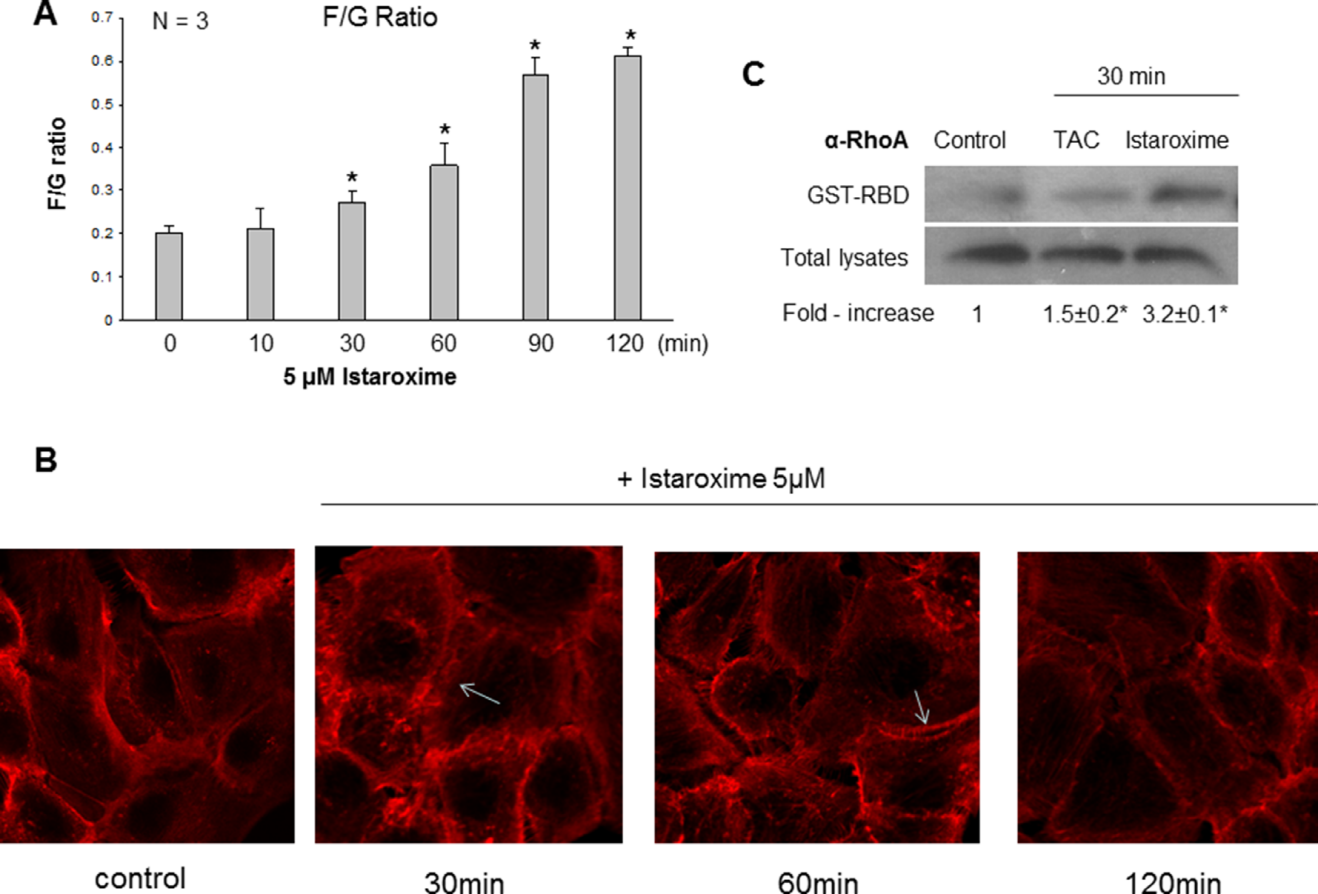
Istaroxime induces actin cytoskeleton re-organization and RhoA activation in prostate cancer cells 24 h serum-starved cells were stimulated with 5 μΜ istaroxime for the indicated time periods (**A**) G- and F- actin were measured by quantitative immunoblot analysis after Triton X-100 subcellular fractionation. Bars present the F/G actin mean value ± SE of *n* = 3 independent duplicate experiments (*P < 0.05). (**B**) Cells treated or not with 5 μΜ istaroxime were stained with rhodamine-phalloidin for filamentous actin and subsequently analysed by confocal laser scanning microscopy. Arrows show stress fibers. Magnification × 100. (**C**) Representative experiment showing the amount of active GTP-bound RhoA determined by a GST pull-down assay, from non-stimulated (control), Testosterone Human Serum Albumin conjugate (TAC, 100 nM)- or istaroxime (5 μM) -stimulated DU145 prostate cancer cells for 30 minutes. RhoA levels were determined by immunoblotting with RhoA antibody in pull-downs (*upper panel*) and total cell extracts (*lower panel*). The immunoblots were analyzed by densitometry, and the intensity of the Rho GTP bands was normalized to the intensity of the corresponding total Rho band. The ratios are presented in fold-increase mean values ± S.E. of the activation of RhoA from three distinct experiments (*P < 0.05).

Recent studies with other CTS have recently reported a reduction of c-Myc oncoprotein expression and actin-cytoskeleton re-arrangements in prostate and lung cancer cells [19, 20]. To assess whether istaroxime induced similar effects, we performed c-Myc Western blot analysis in DU145 prostate cells treated with 5 μΜ of the compound in a time course extending up to 6 hours. In agreement with previous observations, istaroxime significantly down-regulated c-Myc protein levels (Figure 3). c-Myc mRNA levels remained unchanged at the same time (data not shown), indicating that istaroxime-mediated effects most likely occurred through protein destabilization. Interestingly, the compound modulated actin polymerization dynamics by inducing rapid -within 30 minutes- F/G-actin ratio increase; actin polymerization persisted for at least 120 minutes (Figure 4A). This finding was supported by laser scanning microscopy analysis (Figure 4B), showing a reorganization of the actin network with formation of stress fibers (Figure 4B arrows) in treated cells.

The Rho family of small GTPases holds a prominent role in regulating rapid actin reorganization induced by various effectors [21–23]. Thus, we performed affinity precipitation assays with a GST-fusion protein comprising the rhotekin Rho-binding domain (GST-RBD) to assess RhoA GTP loading [24]. As shown in Figure 4C, treatment with a positive control (PC; 10^−7^ M testosterone-HSA conjugate) induced a rapid and moderate 1.5 fold activation of RhoA as reported previously [14, 25]. Interestingly, istaroxime was also able to induce early and robust RhoA activation (3.2 fold); this result was in agreement with its capacity to induce rapid actin polymerization and the reported role of RhoA in this process [23, 26].

### Istaroxime precludes binding of testosterone conjugates to the membrane androgen receptor

**Figure 5:**
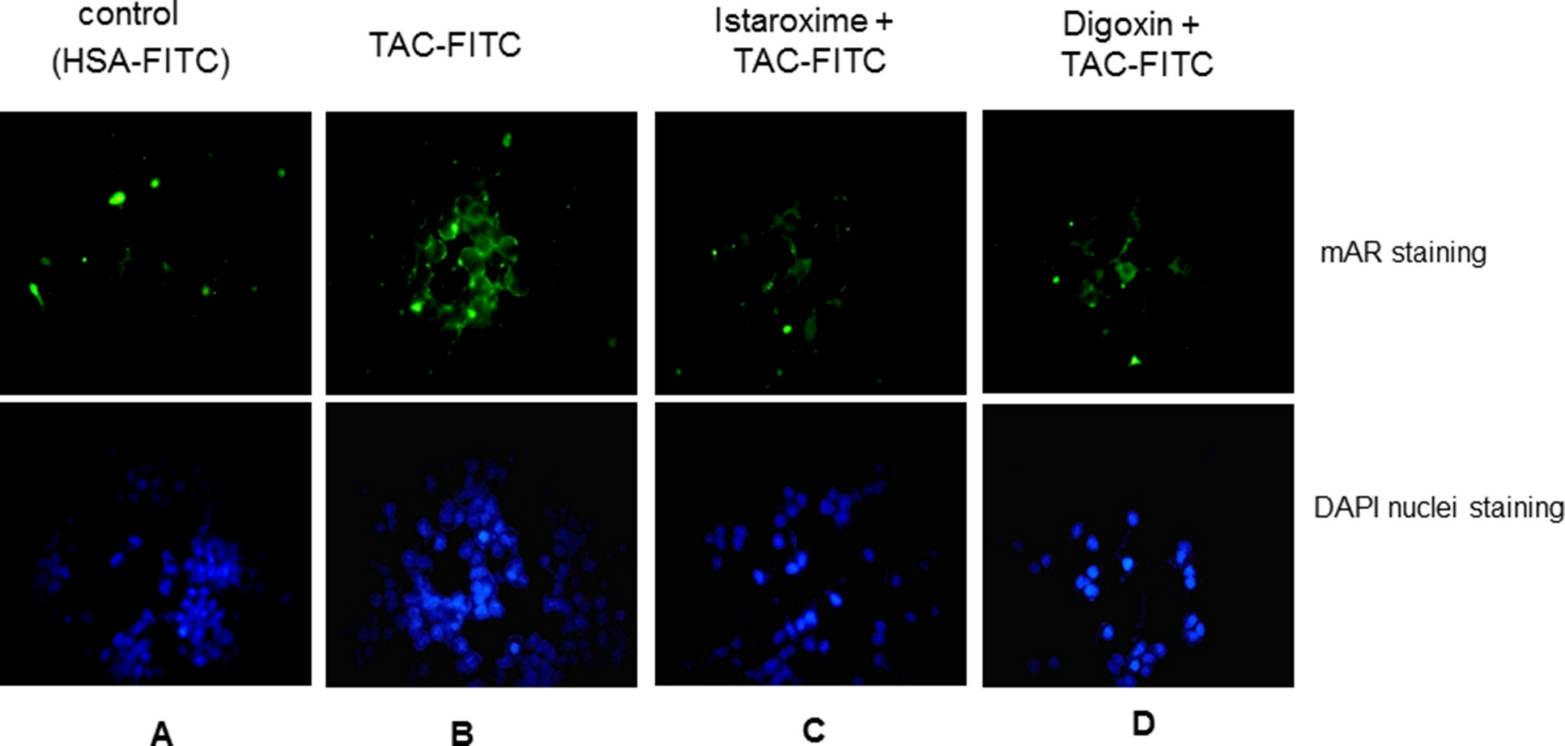
Istaroxime and digoxin prohibit association of testosterone conjugates to the membrane androgen receptor Fluorescence microscopic analysis of LNCaP prostate tumor cells stained with (**A**) HSA-FITC (control) showing no apparent membrane fluorescence, (**B**) testosterone-HSA (TAC)-FITC, showing specific FITC related fluorescence at the cell membranes, (**C**) and (**D**) cells pre-incubated with 5 μM istaroxime and digoxin, respectively, before staining with testosterone-HSA-FITC showing no apparent membrane fluorescence staining. Visualization of nuclei was evident by DAPI. Magnification, × 20.

Results reported above indicated a major role of istaroxime in actin cytoskeleton re-organization and rapid RhoA activation in prostate cancer cells. Since: i) similar properties are shared by ligands of the membrane androgen receptor (mAR) (e.g. Testosterone-Human Serum Albumin conjugates; Testo-HSA conjugates, TAC), [14, 15] and ii) istaroxime contains the typical steroid A-D ring structure similar to testosterone-HSA conjugates (Figure 1A), we tested the capacity of this compound to preclude binding of a fluorescent Testosterone-HSA conjugate (TAC-FITC) to mAR in fluorescent-based assays as described previously [27]. For these assays, we used LNCaP prostate cancer cells due to their optimal mAR detection properties and similarities in growth responses to istaroxime in comparison to DU145 or PC-3 cells (data not shown). In agreement with published results [27], TAC-FITC conjugates showed clear membrane staining, (Figure 5, Panel B) while cells stained with empty HSA-conjugates (HSA-FITC) exhibited no membrane staining (Figure 5, Panel A). Pre-incubation of cells with istaroxime resulted in loss of membrane fluorescence indicating that istaroxime precludes binding of TAC-FITC to the membrane androgen receptor (Figure 5, Panel C). Similar results were obtained with another Na^+^/K^+^ ATPase inhibitor, digoxin (Figure 5, Panel D).

### Istaroxime displaces radioactive testosterone bound to the membrane androgen receptor

**Figure 6:**
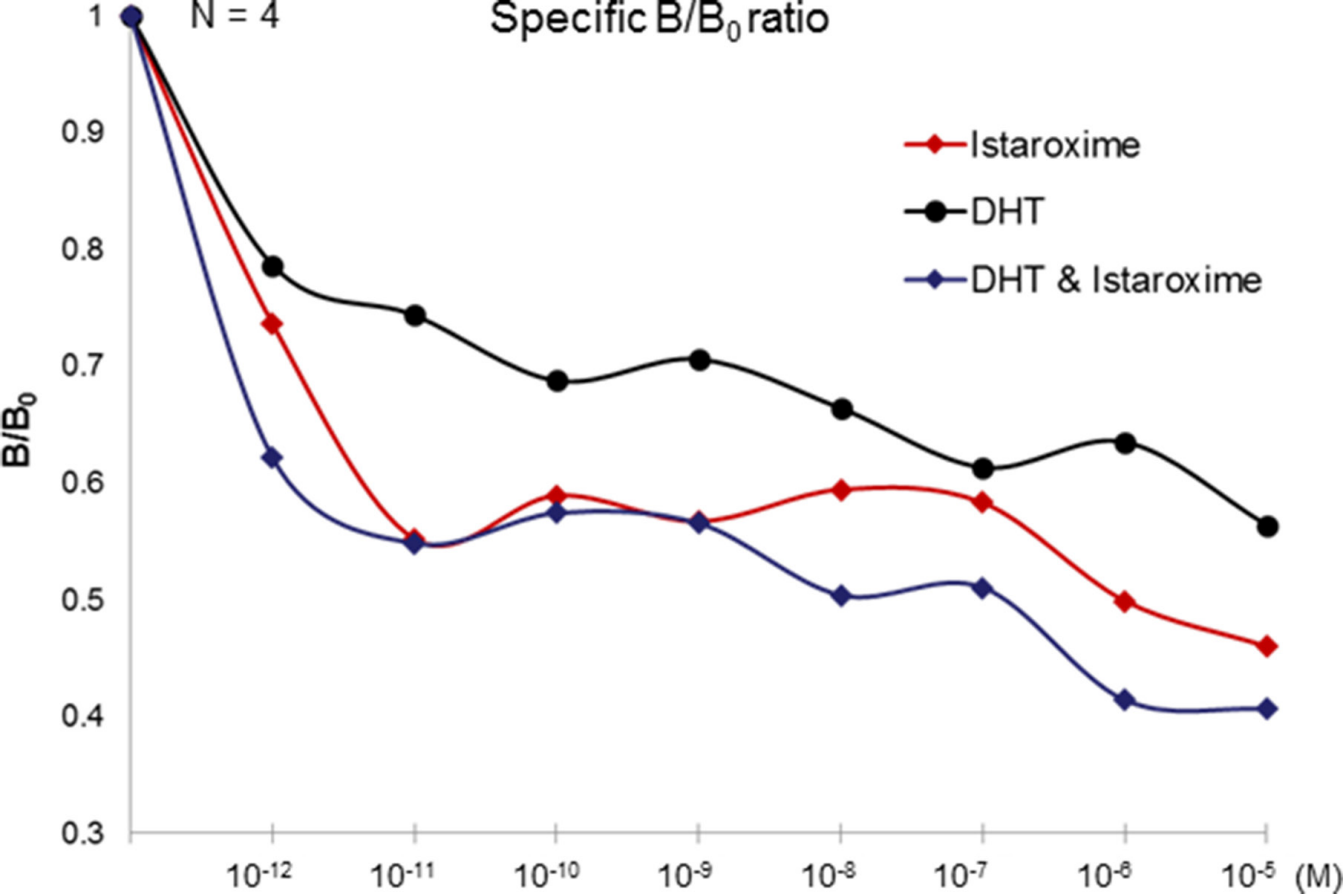
Displacement binding assay Cell membranes of DU145 cells were incubated with 5 nM of [3H] testosterone alone (Bo) or in the presence of the indicated concentrations of unlabelled steroids in the presence or absence of istaroxime (DHT, DHT + Istaroxime), ranging from 10^−12^ to 10^−5^ M. Nonspecific binding was assayed by introducing 5 μM DHT. The Figure (representative Figure from *n* = 3 different experiments performed in duplicate) presents the ratio of specific binding in the presence of the indicated concentrations of DHT or DHT + Istaroxime (B) to the specific binding in the absence of DHT (Bo), B/Bo.

To further characterize the putative interaction of istaroxime with mAR, we performed binding experiments using radiolabeled testosterone in membrane preparations of DU145 cells. As shown in Figure 6, membrane preparations incubated with [^3^H]-testosterone in the presence of increasing concentrations of Dihydrotestosterone (DHT) (10^−12^ to 10^−5^ M) revealed a displacement of radiolabeled testosterone by DHT. In agreement with fluorescent assay results, istaroxime displaced radiolabeled testosterone and was more potent than DHT in this assay. Displacement of radiolabeled testosterone seemed to be more pronounced when both DHT and istaroxime were combined. However, differences between groups were not statistically significant. These results indicated direct binding of istaroxime to the mAR. Of note, similar results were obtained with digoxin (data not shown).

## DISCUSSION

Recent preclinical and clinical data point to a potential anti-cancer effect of Na^+^/K^+^ ATPase inhibition in various indications [28]. For example, several cancer types harboring isoform overexpression or holoenzyme subunit alterations have been identified (reviewed in [11]; references herein). In addition, a series of screening and epidemiological studies qualify CTS as highly active anti-cancer compounds with the potential to reduce cancer risk [29–33]. In agreement with these observations, plant derived inhibitors such as PBI-05204/oleandrin or HuaChanShu have shown anti-cancer activity in early clinical trials [34–36].

In this study, we focused on istaroxime, a Na^+^/K^+^ ATPase inhibitor designed to overcome known pro-arrhythmic and other limitations of CTS [6, 18]. Having shown favorable properties in cardiac phase II clinical trials [8–10], istaroxime seemed an ideal cancer re-purposing candidate. Our growth assays in multiple cell lines (Table 1) together with efficacy experiments in PC-3 prostate cancer xenografts *in vivo* (Figure 1) confirmed the purported anti-cancer effects of this compound. Analyzing istaroxime's mechanism of action, we have shown that the compound induced apoptosis and caspase-3 activation in prostate cancer cells (Figure 2). Similar to what was shown for other Na^+^/K^+^ ATPase inhibitors, istaroxime suppressed c-Myc oncoprotein expression (Figure 3) and induced actin cytoskeleton re-organization in prostate cancer cells (Figure 4). Finally, in agreement with its actin re-modeling role, istaroxime treatment resulted in rapid activation of RhoA signaling (Figure 4C). We believe that the activation of RhoA induced by istaroxime is an early and transient stimulus of actin polymerization, rather than associated with enhanced invasiveness of the cells [37]. In line with this, a wound healing assay showed clear inhibition of cell migration in DU145 cells treated by istaroxime as compared to untreated cells (data not shown). Further studies are now needed to address this issue in more detail.

Searching for additional targets and/or downstream effectors of istaroxime, we have also tested the potential functional crosstalk of Na^+^/K^+^ ATPase with mAR, a membrane receptor mediating rapid, non-genomic anti-cancer effects of androgens [14, 15]. Our hypothesis was triggered by the observation that mAR activation shared several similarities to Na^+^/K^+^ ATPase inhibition, namely induction of rapid actin cytoskeleton reorganization and rapid RhoA activation (Figure 4; [15]). Using both fluorescent binding exclusion (Figure 5) and displacement assays (Figure 6), we have shown binding of istaroxime to the mAR. This indicated functional crosstalk of this receptor with the Na^+^/K^+^ ATPase.

These results initially supported the idea that the membrane pool of Na^+^/K^+^ ATPase may actually serve as the elusive mAR, a hypothesis also put forward more than 20 years ago by Farnsworth [38]. However, several lines of evidence argue against this hypothesis. First, we have been unable to show direct inhibition of Na^+^/K^+^ ATPase activity by testosterone, DHT, testosterone-albumin conjugates or testosterone 3-CMO (a selective mAR ligand [39]) in assays using purified Na^+^/K^+^ ATPase (data not shown). Similarly, assays in Xenopus laevis oocytes failed to show any influence of testosterone on Na^+^/K^+^ ATPase currents (data not shown). Third, neither istaroxime nor digoxin or ouabain were capable of blocking mAR dependent apoptosis triggered by testosterone-albumin conjugates in prostate cancer cells and vice versa (data not shown). These results together with observations that mAR is a G-protein coupled receptor (reviewed in [40]) further support the notion that Na^+^/K^+^ ATPase and mAR may not be identical. Indeed, the finding that istaroxime together with DHT was more effective than each agent alone in displacement assays performed with membranes of DU145 cells (Figure 6), support the notion that Na^+^/K^+^ ATPase and mAR may either share a common subunit, be part of a bigger membrane complex or be functionally interlinked. For example, it is possible that istaroxime binds to a Na^+^/K^+^ ATPase accessory factor increasing its affinity to mAR or a relevant complex. This may result in mAR activation (explaining the results on rapid RhoA activation and actin re-organization, Figure 4). Alternatively, membrane calcium channels could be formed on the membrane linking Na^+^/K^+^ ATPase and mAR [41]. This could explain the observations that both, Na^+^/K^+^ ATPase inhibition and mAR activation result in an increase in intracellular Ca^2+^ levels [42–44].

The pore forming Ca^2+^ release activated Ca^2+^ channel (CRAC) subunit Orai, shown to participate in both mAR signaling and regulation of Na^+^/K^+^ ATPase expression in various tumors [45–48] may play as well a role. The same applies for Na^+^/H^+^ exchanger and glucocorticoid inducible kinase-1 (SGK1), both shown to be functionally interlinked with mAR and Na^+^/K^+^ ATPase [49–52]. Additional candidates include GPRC6A, a Ca^2+^ activated G-protein coupled receptor proposed to be identical to mAR [53, 54] or the zinc transporter ZIP9 also proposed to fulfill the role of mAR in cancer cells [55]. Finally, it is worth noting that potential involvement of the intracellular androgen receptor (iAR) to Na^+^/K^+^ ATPase is highly unlikely at this point since i) iAR is functionally discrete to mAR as shown by multiple publications (reviewed by [15]) and ii) istaroxime does not bind the iAR [6]. On the other hand, published observations that progesterone may directly bind the Na^+^/K^+^ ATPase complex clearly point to the necessity of performing a more detailed functional characterization of the interplay of Na^+^/K^+^ ATPase with mAR, iAR or other steroid receptors.

In conclusion, istaroxime, a clinically validated cardiac Na^+^/K^+^ ATPase inhibitor, exhibits strong anti-cancer potential *in vitro* and in *in vivo*. Our results and reported mechanisms of action support a drug re-purposing potential of the compound against prostate and other tumors.

## METHODS

### Compounds

Istaroxime was synthesized as described previously [6] with modifications (see Supplementary Methods). DHT, HSA-FITC, Testosterone-HSA, Testosterone-HSA-FITC conjugates, Testosterone-3-CMO, ouabain and digoxin were obtained by Sigma (Germany).

### Ethical statement

Investigation has been conducted in accordance with the ethical standards and according to the Declaration of Helsinki and according to national and international guidelines and has been approved by the authors’ institutional review board.

### Cell lines

With the exception of CCSWD1 cells (generated from a human clear cell sarcoma/malignant melanoma tumor [56]), and normal human fibroblast cells (Lonza, USA), all cancer lines were obtained from the American Type Culture Collection (Manassas, VA) or the National Cancer Institute (NCI), NIH (Bethesda, MD, USA). These cell lines are categorized as follows: lung: A549, EKVX, H460; melanoma: SK-MEL28, LOXIMVIb, MDA-MB-435; breast: MCF7, T47D; ovarian: OVCAR-5, OVCAR-3, IGROV1, NCI-ADRES; prostate: PC-3, DU145, LNCaP; pancreas: SU8686; renal: CAKI-1; Central Nervous System: SF-295, U251, SF-268; colon: HCT-116, HCT15. Cell lines were adapted to grow in RPMI1640 supplemented with 25 mM HEPES, 2 mM L-Glutamine, 5–10% fetal bovine serum and antibiotics in a 5% CO_2_ humidified atmosphere at 37°C.

### Na^+^/K^+^ ATPase assays

Istaroxime's inhibitory effect on ATPase activity was assessed *in vitro* using the Adenosine 5′-Triphosphatase Enzymatic Assay of Sigma (St.Louis, MO) according to the manufacturer's instructions as reported previously [12]. This assay utilizes enzyme isolated from porcine cerebral cortex.

### MTT and SRB assays

Cell proliferation/viability was assessed by MTT [3-(4, 5-dimethylthiazol-2-yl)-2, 5-diphenyltetrazolium bromide] assays (Sigma, St.Louis, MO) done as previously described [12]. Sulforhodamine B (SRB) assays were performed according to NCI guidelines for anti-cancer drug screening (https://dtp.cancer.gov/discovery_development/nci-60/default.htm) and as previously published [12, 57]. In SRB assays, three dose response parameters were calculated for each agent. Growth inhibition of 50% (GI_50_): drug concentration resulting in a 50% reduction of growth; Total growth inhibition (TGI): drug concentration resulting in a 100% reduction of growth and lethal concentration of 50% (LC_50_): concentration of drug resulting in a 50% reduction of cell viability. Typically, all assays were done in triplicates in 2–3 repetitions for each compound. This is deemed sufficient to extrapolate on the activity of each compound. Note that MTT-calculated IC_50_ values may differ from SRB-assay calculated GI_50_ values due to inherent differences in assay methodologies.

### Maximum tolerated dose (MTD) assays

Female NOD/scid mice, 8 weeks old, weighing ~21 g were used for the MTD studies according to NCI guidelines (https://dtp.cancer.gov/organization/btb/acute_toxicity.htm). Briefly, one animal per individual dose was injected with a single dose of 200/100/50/25/12.5 mg/kg of instaroxime respectively, in a volume of 20 μL/g of weight. Animals were weighed prior to each administration and volumes/dose administered was adjusted according to body weights. The animal that received 200 mg/kg suffered from sedation but recovered within 1–2 hours; all other animals showed no side effects. In a second round of experiments, animals received once-daily doses of 40 and 50 mg/kg for several consecutive days; these treatments were also well tolerated. Based on these studies, we concluded that the compound's acute MTD was 200 mg/kg whereas the chronic MTD was > 50 mg/kg.

### PC-3 xenograft studies

Xenografts were generated by subcutaneously injecting exponentially growing cultures of ~2 × 10^6^ PC-3 cells (in Matrigel in 0.1 ml PBS) at the right flank of 6–8 weeks old male Balb/c nude mice. Following development of palpable tumors (150–200 mm^3^) and group randomization (10 animals per group), the following treatments were administered:

Group A: Water for Injection (WFI), IP, twice daily for 23 days at 12 hours intervals.

Group B: Istaroxime at 22.5 mg/kg, IP, twice daily for 23 days at 12 hours intervals.

Group C: Docetaxel at 12 mg/kg, IV, once weekly for 23 days.

Tumor volumes were measured twice weekly in two dimensions using a caliper according to the formula V = 1/2 × a × b^2^ where a and b are the long and short diameters of the tumor respectively. %DT/DC values were also calculated, where DT = T − Do and DC = C − Do (Do is the average tumor volume at the beginning of the treatment; T and C are the volumes of treated and untreated tumors, respectively, at a specified day). Tumor Growth Inhibition was calculated as the percentage of tumor volume versus vehicle control in a given date. Losses of weight, neurological disorders, behavioral and dietary changes were also recorded as indicators of toxicity (side effects). Experiments were terminated when tumors in control animals reached a volume of ~2000–2500 mm^3^ (about 11% of the body weight).

### Ethical conduct of animal experiments

MTD experiments were performed at the Pharmacy Department, University of Athens, Greece under the approval of the veterinary committee (Approval number Κ/2844) and in agreement with Greek laws (2015/92), EU & European council guidelines (86/609 and ETS123, respectively), and Compliance with Standards for Human Care and Use of Laboratory Animals, NIH, USA (Assurance No. A5736–01).

Animals used in xenograft studies were treated according to the Guide for the Care and Use of Laboratory Animals, Institute of Laboratory Animal Resources, National Academy Press, Washington, 1996. Specific conditions regarding handling of moribund animals as determined by the veterinary staff of the test facility were explicitly defined in the study protocol according to international guidelines including euthanasia for humane reasons. This included carbon dioxide inhalation followed by exsanguination. Final disposition of all animals placed on study was documented in all study records.

### TUNEL apoptosis assay and FACS analysis

DU145 cells were cultured in serum containing medium in the absence or presence of 5 μM istaroxime for 24 hours. At the end of the treatment, cells were harvested in PBS and apoptosis was assessed using the APO-BrdU^™^ TUNEL Assay kit (Molecular Probes, Eugene, OR) according to the manufacturer's instructions. The APO-BrdU^™^ TUNEL Assay kit labelled DNA strand breaks for the detection of apoptotic cells through addition of BrdU at the 3′-hydroxyl ends by terminal deoxynucleotide transferase. Final detection of BrdU incorporation at DNA break sites was detected using an Alexa Fluor488–labeled anti-BrdU antibody. Propidium Iodide was used to stain the total cell population. Flow cytometry was performed with FACSArray apparatus (BD Biosciences) and the results were analyzed by the CellQuest software (BD Biosciences).

### Caspase-3 assay

The activity of caspase-3 was measured in whole cell lysates pretreated or not with 5 μM istaroxime for the time periods indicated in the Figure legends, using the Clontech ApoAlert^®^ Caspase Colorimetric Assay kit according to the manufacturer's instructions. Caspase-3 activity was determined by incubating lysates with a caspase-3 substrate (the peptide DEVD conjugated to the chromophor p-nitroaniline) for 2 h at 37^°^C. The absorbance of each sample was measured at 405 nm by using a 96-well colorimetric plate reader.

### Immunoblotting

Cells were incubated with 5 μM istaroxime for the indicated time periods, washed twice with ice cold PBS and suspended in 500 μl ice-cold lysis buffer (50 mM Tris/HCl, 1% TritonX-100 pH 7.4, 1% sodium deoxycholate, 0.1% SDS, 0.15% NaCl, 1 mM EDTA, 1 mM sodium orthovanadate) containing protease inhibitor cocktail (Sigma). The protein concentration was determined using the Bradford assay (BioRad). Sixty μg of protein were solubilized in sample buffer, resolved by 10% SDS-PAGE and transferred on PVDF membranes. The membranes were initially analyzed with anti c-Myc antibody (1:100, 9E10, Santa Cruz Biotechnology, CA) and subsequently, after stripping, with anti-beta Actin (1:100, Santa Cruz Biotechnology, CA). Appropriate horseradish peroxidase conjugated secondary antibodies (1:2000, Cell Signaling, USA) and the ECL detection reagent (Amersham, Germany) were used. Blots were quantified using Quantity One Software (Biorad, Germany).

### Measurement of F/G actin ratio by Triton X-100 fractionation

The Triton X-100 soluble G-actin and Triton X-100 insoluble F-actin containing fractions of cells exposed to 5 μM istaroxime for the time periods indicated in Figure legend, were prepared as previously described [58]. An increase of the triton-insoluble (F-) to the triton-soluble (G-) actin ratio is indicative of actin polymerization.

### Confocal laser scanning microscopy

Cells were cultured on glass coverslips with 5 μM istaroxime for the time points indicated in the Figure legend. For direct fluorescence microscopy of F-actin, cells were fixed with 3% paraformaldehyde in PBS for 30 min, permeabilized with 0.5% Triton X-100 in PBS (10 min) and incubated with rhodamine-phalloidin (Molecular Probes, Eugene, OR, 1:100 dilution) for 40 min in the dark. Confocal microscopy was performed with a Zeiss LSM 5 EXCITER confocal laser-scanning module (Carl Zeiss) and images were analyzed with the instrument's software.

### RhoA activity determination

To determine the activity of RhoA GTPase in testosterone-BSA and istaroxime treated versus untreated cells, affinity precipitation with Rho Assay reagent (GST-RBD) (Upstate) was performed, according to the manufacturer's instructions. Briefly, cells were washed twice in ice-cold TBS and lysed in Mg^2+^ lysis buffer (25 mM HEPES, pH 7.5, 150 mM NaCl, 1% Nonidet P-40, 10 mM MgCl_2_, 1 mM EDTA, 10% glycerol, 25 mM NaF, 10 μg/ml aprotinin, 10 μg/ml leupeptin, 1 mM Na_3_VO_4_). Cleared cell lysates were incubated with 30 μg GST-RBD for 45 min at 4°C. Precipitates were washed three times with Mg^2+^ lysis buffer and suspended in Laemmli's buffer. The precipitates and equal volumes of total protein extracts were analyzed by immunoblotting with mouse monoclonal anti-RhoA (26C4, 1:100, Santa Cruz Biotechnology) as described above.

### Membrane androgen receptor competition assays

To determine the capacity of a given compound to preclude binding of fluorescent testosterone-HSA conjugates (TAC-FITC) to the mAR, starved LNCaP cells grown on coverslips were pre-treated with 40 μΜ of the indicated drug for 30 minutes prior to the addition of testosterone-HSA-FITC. At the end of the incubation period, cells were washed twice with PBS and 40 μΜ of testosterone-HSA-FITC was added. Specimens were prepared and analyzed as described above. Compounds binding to the mAR preclude binding of fluorescent testosterone-HSA conjugates to their target and abolish membrane-specific fluorescence.

### Detection of membrane androgen receptors and competition assays

For membrane preparation DU145 cells cultured in five 75 cm^2^ flasks, were washed twice with PBS, removed by scraping, and centrifuged at 1, 500 g for 5 min. Pelleted cells were homogenized by sonication in 50 mM Tris-HCl buffer, pH 7.4, containing freshly added protease inhibitors (10 μg/ml PMSF and Roche complete protease inhibitor tablets). Unbroken cells were removed by centrifugation at 2, 500 g for 15 min. Membranes were obtained by centrifugation at 20, 000 g for 1 h and washed once with the same buffer. Protein concentration was measured by the method of Bradford using reagents from Bio-Rad (Hercules, CA). Displacement binding experiments were performed as previously described [59]. In brief, cell membrane preparations at a final concentration of 1.0 mg/ml were incubated with 5 nM [^3^H] testosterone in the absence or in the presence of different concentrations of unlabeled steroid (istaroxime, DHT, istaroxime and DHT), ranging from 10^−12^ to 10^−5^ M. Non-specific binding was estimated in the presence of 5 μM istaroxime. In both types of binding experiments, after an overnight incubation at 4°C, bound radioactivity was separated by filtration under reduced pressure through GF/A filters previously soaked in 0.5% polyethylenimine (PEI) in water and rinsed three times with ice-cold Tris-HCl buffer. Filters were mixed with 10 ml scintillation cocktail (03999, Fluka), and bound radioactivity was counted in a scintillation counter (TriCarb^®^ 2900TR Liquid Scintillation Analyzer, PerkinElmer).

### Statistics

Statistical analysis was performed using the GraphPad Prism software (GraphPad Software, Inc., La Jolla, CA, USA).

## SUPPLEMENTARY MATERIALS


