# Supplementary material for: Functional characterization and anti-cancer action of the clinical phase II cardiac Na+/K+ ATPase inhibitor istaroxime: in vitro and in vivo properties and cross talk with the membrane androgen receptor
Source: Oncotarget. 2016 Mar 24;7(17):24415–28. doi: 10.18632/oncotarget.8329 (PMC5029711; doi:10.18632/oncotarget.8329)
Supplement: Supplementary file 1 [file oncotarget-07-24415-s001.pdf]

Functional characterization and anti-cancer action of the clinical phase II cardiac Na^+/K^+ ATPase inhibitor istaroxime: *in vitro* and *in vivo* properties and cross talk with the membrane androgen receptor

Supplementary Materials

Synthesis of (*E,Z*) 3-(2-aminoethoxyimino)-5*α*-androstane-6, 17-trione (**4**)

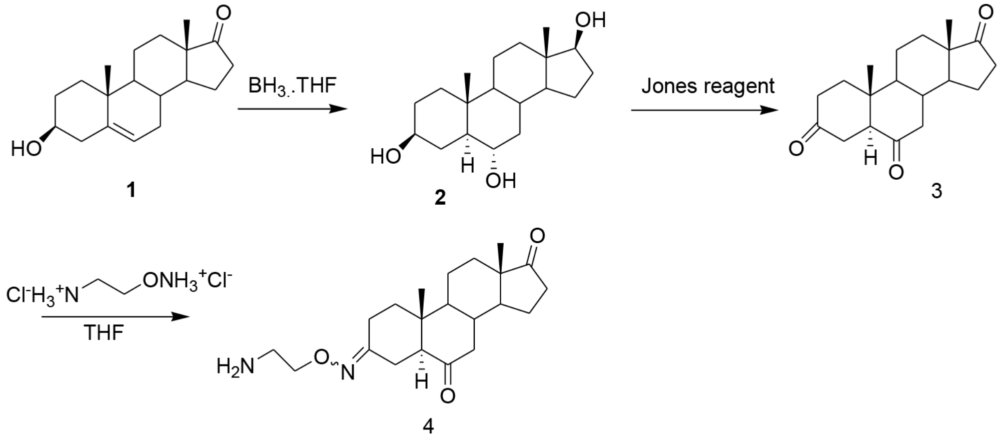

Chemistry

Starting materials and reagents were obtained from commercial suppliers and used without further purification. ^{1}H NMR spectra were recorded at 600 or 300 MHz. ^{13}C NMR spectra at 75 MHz. ^{1}H and ^{13}C NMR spectra are internally referenced to residual solvent signals ($CDCl_3$, CD_3OD, DMSO-d_6). Data for ^{1}H NMR are reported as follows: chemical shift (δ ppm), multiplicity (s = singlet, bs = broad singlet, m = multiplet), and integration. Melting points (°C) are uncorrected. Thin-layer chromatography (TLC) was performed on glass plates coated with silica gel (0.2 mm, 60 F254) and flash column chromatography using silica gel (200–400 mesh). HR-MS spectra were obtained using a UHPLC-MS^n Orbitrap Velos-Thermo mass spectrometer.

5α-Androstane-3β,6α,17β-triol (2)

To a stirred solution of dehydroepiandrosterone (1) (5.0 g, 17.34 mmol) in THF (75 mL) at –10°C under N_2 was added 1M BH_3•THF complex in THF (44 mL, 44 mmol). After completing the addition, the bath was removed and the mixture was stirred at room temperature for 5 h. The reaction was cooled to 0°C and H_2O (85 mL) was cautiously added dropwise, followed by $NaBO_3$•$4H_2O$ (5.23 g, 34 mmol). After stirring at room temperature overnight, the solid was filtered, washed with THF (3 × 50 mL) and then discarded. The organic phase was separated and the aqueous phase was saturated with NaCl and extracted with THF (3 × 50 mL). The combined organic extracts were dried over Na_2SO_4, filtered, and evaporated in vacuo to dryness. The crude product was crystallized from EtOAc/MeOH (2/1, 10 mL/g) to give a first crop of compound 2 as a white solid (2.7 g). The mother liquors were evaporated and the residue crystallized from EtOAc/MeOH (2/1, 10 mL/g) to give a second crop of compound 2 as a white solid (0.94 g). The procedure was repeated to give a third crop of compound 2 as a white solid (0.37 g); (In total 4.01 g; 75% overall yield).

Mp: 232–234°C; Rf: 0.42 (CH_2Cl_2:MeOH, 9:1); ^{1}H NMR (600 MHz, $CDCl_3$): *δ* 3.56 (m, 1H), 3.46 (m, 1 H), 3.30 (m, 1H), 2.16 (m, 1H), 2.00–0.90 (m, 17H), 0.83 (s, 3H), 0.81 (m, 1H), 0.71 (s, 3H), 0.66 (m, 1H); ^{13}C NMR (75 MHz, CD_3OD): *δ* 82.5, 72.0, 70.0, 55.6, 53.0, 52.3, 44.2, 42.3, 38.7, 38.0, 37.5, 35.8, 33.1, 31.9, 30.7, 24.4, 21.9, 13.9, 11.7; HRMS (ESI^+): $[M+Na]^+$, found 331.2240. $C_{19}H_{32}O_3Na$ requires 331.2244.

5α-Androstane-3, 6,17-trione (3)

To a solution of 5α-androstane-3β,6α,17β-triol (2) (2.9 g, 9.4 mmol) in acetone (74 mL) was added an excess of Jones reagent (11 mL) dropwise, maintaining the temperature below 35°C. 5 min after completion of the addition, the excess of Jones reagent was neutralised by addition of *i*-PrOH (13.5 mL) and after further 10 min stirring, the suspension was filtered from a short silica pad and the filtrate evaporated to dryness. The residue was treated with H_2O (50 mL) and extracted with EtOAc (3 × 60 mL). The combined organic extracts were washed with 5% aqueous $NaHCO_3$ solution (50 mL), H_2O (50 mL), dried over Na_2SO_4 and evaporated to dryness. The residue was purified by flash column chromatography (elution solvent, DCM:MeOH, 98:2) to give 5α-androstane-3, 6,17-trione (3) as a white solid (2.1 g, 74%).

Mp: 195–197°C. (lit:10b 196–196.5°C); Rf: 0.87 (CH_2Cl_2:MeOH, 9:1); ^{1}H NMR (600 MHz, $CDCl_3$): δ 2.70–1.30 (m, 20H), 0.99 (s, 3H), 0.91 (s, 3H); ^{13}C NMR (75 MHz, $CDCl_3$): δ 219.3, 210.6, 208.0, 57.4, 53.3, 51.4, 47.9, 45.2, 41.0, 37.9, 37.3, 37.2, 36.8, 35.5, 30.9, 21.5, 20.8, 13.7, 12.5; HRMS (ESI$^+$): $[M^+H]^+$, found 303.1952. $C_{19}H_{27}O_3$ requires 303.1955.

(E, Z) 3-(2-Aminoethoxyimino)-5α-androstane-6, 17-trione (4)

To a stirred solution of 5α-androstane-3, 6,17-trione (3) (1.83 g, 6.05 mmol) in THF (40 mL), a solution of the 2-aminoethoxyamine dihydrochloride (894 mg, 6 mmol) (Pankaskie and Scholtz 1989) in H_2O (19 mL) was rapidly added dropwise. After 1.5 h, NaCl (2.63 g, 45 mmol) was added and the mixture was stirred for 10 min. The layers were separated and the aqueous layer was extracted with THF (3 × 50 mL). The combined organic extracts were dried over Na_2SO_4, filtered, and evaporated to give an oily residue. The crude product was dissolved in CH_2Cl_2 (0.15 M) and washed with a saturated aqueous solution of NaCl (3 × 30 mL). The organic layer was dried again over Na_2SO_4 and evaporated to dryness. The crude product was crystallized from MeOH/EtOAc to afford the HCl salt of (E, Z) 3-(2-aminoethoxyimino)-5α-androstane-6, 17-trione (4), 1.33 g 56% yield as a white solid.

Mp: 220–225°C (dec); Rf: 0.37 (CH_2Cl_2:MeOH, 9:1); ^{1}H-NMR (600 MHz, DMSO-d_6): δ 9.38 (2H, bs), 4.73 (1H, m), 3.35–2.90 (5H, m), 2.60–1.15 (21H, m), 0.79 and 0.76 and 0.76 (s and s and s, 6H); ^{13}C NMR (75 MHz, CD_3OD): δ 222.6, 212.0, 211.9, 162.0, 161.9, 70.07, 70.05, 58.6, 57.4, 54.4, 54.3, 52.6, 49.9, 49.4, 49.3, 48.2, 46.1, 46.0, 42.7, 42.6, 40.6, 40.5, 38.9, 38.7, 38.6, 37.9, 36.6, 32.4, 27.9, 27.8, 22.6, 21.9, 21.8, 21.6, 14.2, 12.7, 12.6; HRMS (ESI$^+$): $[M+H]^+$, found 361.2479. $C_{21}H_{33}N_2O_3$ requires 361.2491.

REFERENCES

1. Pankaskie MC, Scholtz SA. An Improved Synthetic Route to Aminoxypropylamine (APA) and Related Homologs. Synth. Commun. 1989; 19:339–344.
